# Leisure Participation Behavior and Psychological Well-Being of Elderly Adults: An Empirical Study of Tai Chi Chuan in China

**DOI:** 10.3390/ijerph16183387

**Published:** 2019-09-12

**Authors:** Jing Li, Chia-Chien Hsu, Ching-Torng Lin

**Affiliations:** 1 Program in Management, Dayeh University, Changhua 51591, Taiwan; shorn2003@gmail.com; 2 College of Music and Movie, Tianjin Normal University, Tianjin 300387, China; 3 Department of Tourism, Shih Hsin University, Taipei 116, Taiwan; hsu127@hotmail.com; 4 Department of Information Management, Dayeh University, Changhua 51591, Taiwan

**Keywords:** elderly adults, psychological well-being, Tai Chi Chuan, theory of planned behavior, technology acceptance model

## Abstract

Theoretical explanation concerning the psychological well-being of elderly adults as they participate in a particular leisure activity has been rare. Based on the theory of planned behavior and the technology acceptance model, this study sought to understand the Tai Chi Chuan (TCC) participation influence factors, process, and psychological well-being of Chinese people. A self-developed questionnaire was developed to test the hypotheses of this study. Applying structural equation models, a survey of 769 TCC participations were used to test the theoretical model. The results indicated that perceived usefulness significantly and positively affect respondent attitude, family members’ influences significantly and positively affect participants’ subjective norm, and resource facilitating conditions significantly and positively affect perceived behavioral control. Perceived behavioral control, attitude, and subjective norm significantly and positively affect TCC participants. The study lends support to the notion that leisure activity participation is vital for elderly adults and their well-being, and it develops a theoretical reference model for better understanding the leisure participation perceptual reasoning processes of elderly adults. Furthermore, the results offer important implications for health policy makers, clinical prevention, and interventions using participation behavior relationships to develop well-constructed leisure programs to attract and inspire participation and, hence, improve the psychological well-being of elderly adults.

## 1. Introduction

The increase in life expectancy and the aging of the baby-boomer generation has changed the population age structure for many countries [1]. The growth in the number of elderly adults, people aged 60 years and older, has created the increases in public expenditures for this age group, and the demand for elderly adult care programs and quality of life services. Living for a long time is both possible and desirable, but the aging trend reflects the reality that elderly adults are having to make adjustment as they learn to deal with additional idle time resulting from retirement. Awareness about the changing circumstances related to aging is subtle and varies by individual. Many individuals may perceive that elderly adults require special attention because of the decline in physical strength, cognitive function, and the narrowing of their social networks. As a matter of fact, the majority of elderly adults are fit, they live independently, and they actively engage in a variety of activities [2,3]. Helping elderly adults’ transition into a different pattern of living while still increasing their quality of life has become a social phenomenon [4]. As such, efforts to identify factors that promote well-being as perceived by elderly adults is of growing importance and worthy of further research efforts. Behaviors that promote well-being such as leisure activity participation and attitudes toward leisure participation, as well as subjective norms.

In the literature, accumulating evidence reveals that leisure activity participation (e.g., sports, cultural activities, voluntary work, reading books, puzzle solving, etc.) is associated with psychological health in advanced age [5,6]. Previous research efforts generally agree that leisure participation and perceived well-being are all positively related [7,8,9]. These research projects were mostly descriptive and correlational in nature [10,11]. Few of these studies were based on well-constructed theoretical models as they studied elderly adults’ decision-making processes involved in a volitional and non-volitional relation while also taking leisure participation factors into consideration. More specifically, a theoretical explanation concerning the formation of elderly adults’ psychological well-being and participation in a particular leisure activity over other alternatives has been rare if not totally absent. As a result, although descriptive and correlational in nature, studies have shown the predictors that have contributed to the relation of leisure activity participation and elderly adults’ psychological well-being; however, they may not have adequately provided theory for health policy makers and/or clinical interventions, so they can build and evaluate well-constructed leisure programs. More specifically, without theoretical reference models to evaluate the perceptual reasoning processes, significant ambiguity exists in construct leisure programs to attract and inspire elderly adults’ participation, and ultimately increase their perceived psychological well-being. As such, the investigation of the underlying factors leading to leisure participation behavior and its relation to psychological well-being in elderly adults should help interventionists devise well-constructed programs for these individuals. 

The theory of planned behavior (TPB) was developed by Ajzen and Madden [12]. Based on the assumption that most conscious behaviors is rational and goal directed, they proposed a causal link between attitudes and behavior mediated by behavioral intentions. Intention directly influences behavior and it is shaped by attitudes, subjective norms and perceived behavioral control regarding the behavior. From it was developed, The TPB is a widely applied socio-cognitive model of the attitude–behavior relationship, including leisure participation behavior [13,14,15]. Similarly, the technology acceptance model (TAM) introduced by Davis [16] has received considerable attention, having become established as a frugal, yet powerful, model for explaining and predicting usage intentions and acceptance behavior [17]. 

Many leisure activities are acceptable for elderly adults, such as practicing Tai Chi Chuan (TCC), taking a walk, reading, puzzle solving, resting activities, helping others, etc. The TCC process originated in China as a form of martial art and exercise, and it is a popular form of physical activity among Chinese population, which began a long time ago [18]. The TCC exercise incorporates a series of weight shifting, body rotations, semi-squat positions and long durations, which equates to brisk walking. However, TCC is a low-impact, slow-motion exercise, which may elicit greater adherence than brisk walking, leading to less attrition [19]. Therefore, TCC is a suitable social culture leisure activity and it is also a training exercise for the prevention of accidental falls among elderly adults [18]. Nowadays millions of Chinese practice TCC, and it has become one of the most popular forms of exercise or sport, especially among elderly adults. Elderly adults practicing TCC are selected as the study sample.

Based on the above discussion, by combining TAM as a part of TPB model, critical constructs (i.e., perceived ease of use, perceived usefulness) were incorporated into the newly developed model. Thus, the aim of the study was to test the applicability of TPB and TAM in explaining Chinese elderly adults’ decision making processes and factors that lead to their leisure participation, and how such leisure participation affects their psychological well-being. The results show that perceived usefulness had significantly and positively affected elderly adults’ attitude, family members’ influences significantly and positively affected participants’ subjective norms, and resource facilitating conditions significantly and positively affected perceived behavioral control. Furthermore, perceived behavioral control, attitude, and subjective norm have significantly and positively affected TCC participation behavior and, ultimately, affected elderly adults perceived psychological well-being significantly and positively. Our primary contribution to the current literature on leisure and successful aging is to lend support for the fact that leisure activity participation is vital for elderly adults and their well-being, so we developed a theoretical reference model to better understand the leisure participation perceptual reasoning processes of elderly adults. We aim to initiate a new theoretical perspective for the field of leisure participation behavior and well-being of elderly adults that may help policy makers and/or clinical interventionists create and employ leisure programs that will attract and inspire participation.

## 2. Literature Review

### 2.1. Theory of Planned Behavior

Ajzen and Fishbein [20] proposed the theory of reasoned action (TRA), which posits that most human behaviors follow a certain pattern and, therefore, become predictable. According to TRA, individuals with a high degree of volitional control are able to make rational decisions. The TRA model is one of the intention-based models that employs behavioral intention for the purpose of predicting subjects’ actual behaviors. Behavioral intention subsequently results in actual behavior and it is considered a function of attitude toward a behavior and toward a subjective norm. Attitude is associated with behavioral belief and subjective norm is related to normative belief [20].

**Figure 1:**
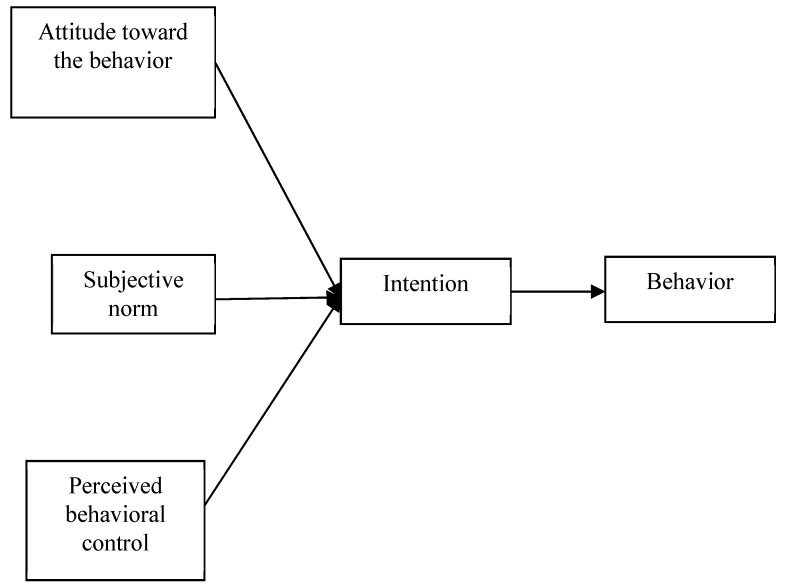
Theory of planned behavior [12].

The TPB, as shown in Figure 1, incorporates the new dimension of perceived behavioral control into TRA as one of the determinants of behavioral intention. Accordingly, this dimension is related to control beliefs. Basically, TPB expands the theoretical framework of TRA, because TRA is unable to fully explain certain situations if the availability of resources and opportunities are limited. As such, addressing the presence of opportunities and resources, which are non-volitional in nature, is important [21]. Actually, TPB mainly offers a basic framework to explain the influence of external variables towards behavioral ideas. Practically, one must cooperate with the theme characteristics to choose different external variables and probe the roles that these variables play.

Evidence suggests that TPB strongly predicts actual human behavior [22]. Thus, TPB has been a widely applied socio-cognitive model of the attitude–behavior relationship, including leisure participation behavior [13,14,15]. In this study, TPB is applied because elderly adults’ decision making process can consist of a variety of non-volitional factors that can either diminish or enhance their opportunities to participate in TCC. Applying TPB as the conceptual framework of this study, researchers are capable of using its well-developed structure to conduct an elaborate examination of the formation of elderly adults’ behavior in TCC and their psychological well-being by considering both volitional and non-volitional factors.

### 2.2. Technology Acceptance Model

**Figure 2:**
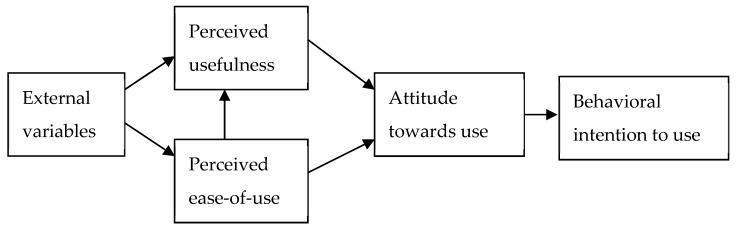
Technology acceptance model [16].

Based on social psychology, TRA was very important to other models as it is one of the most fundamental and influential theories of human behavior. The TRA asserts that both the attitude towards a specific behavior and subjective norm impact behavioral intention, which, in turn, determines actual behavior. Sharing the common thread with TRA, the TAM, as shown in Figure 2, developed by Davis [16] and originally applied in the information system field, is an attitudinal model to explain the effect of system features on user acceptance. Davis [16] notes that individual attitude toward using a particular system or device is a function of two major factors, perceived ease of use and perceived usefulness. However, TAM deviated from TRA from the start, by leaving subjective norms out of the model. In the attitudinal studies, the attitudes are typically defined as learned predispositions that react to a particular object or objects in a favorable or an unfavorable manner [23]. The core idea in studying attitudes in this way corresponds to an individual’s attitude toward a particular object or objects and, subsequently, the changes in behaviors that result from those attitudes [24]. In correlational terms, when a study reveals that attitudes and behaviors are highly correlated, the behavior of an individual is likely to become predictable because his/her attitude toward that specific object has been readily identified and becomes foreseeable. 

### 2.3. Psychological Well-Being

The concept of life satisfaction or overall well-being is basically determined by assessments of individual life concerns [25]. This concept can be considered as a cognitive and judgmental process and an evaluation of a person’s quality of life based upon his/her chosen evaluation criteria [26]. As a cognitive component, psychological well-being is usually viewed as a part of a global perception of life satisfaction or overall well-being perceived by an individual [7]. Winefield et al. [27] indicate that psychological well-being is conceptualized as a mix of positive affective state (e.g., happiness, satisfaction) and functioning with optimal effectiveness in life (e.g., private life, social life). Huppert [28] simply notes that “psychological well-being is about lives going well. It is the combination of feeling good and functioning effectively” (p. 137). That is to say, if an individual perceives a higher degree of psychological well-being, he/she may be happy, confident, and satisfied with life. Additionally, he/she is likely to receive strong support from family members, friends, or co-workers [27]. 

Psychological well-being is considered as a subjective measure of one’s life. The subjective nature reveals problems in terms of measuring psychological well-being because some individuals may focus on a particular life domain (e.g., leisure) while other individuals may emphasize a different life value (e.g., social relationship). It is generally agreed that the focus of the construct of psychological well-being is on the positive aspects of individual perception. However, the measurement of psychological well-being has not enjoyed a universal standard of acceptance [27]. The scale of subjective well-being used by Xu and Roberts [29] include the following three dimensions: global life satisfaction, domain life satisfaction, and positive affect. Ryff and Keyes [30] categorize psychological well-being into the following six dimensions: self-acceptance, positive relations with others, autonomy, environmental mastery, purpose of life, and personal growth. As such, the key to measuring psychological well-being lies in the fact that researchers can be hard pressed to know the cutoff points between “too much” and “too little” concerning each person’s perception, preferences, and individual needs [31].

### 2.4. Leisure Participation Behavior

Leisure is an essential domain or characteristic of life and, concomitantly, the measurement of the quality of life as well. It balances individual life rhythm and it can influence one’s health. Engaging in leisure activities, also called leisure participation behavior, is able to help reduce stress, depression, and loneliness [32,33]. In the literature, the importance of participation in a variety of leisure activities has been well-documented [32]. From the social exchange perspective, individuals participate in a leisure activity with the expectation of receiving some sort of reward or benefit. Searle [34] indicates that participants of a leisure activity will discontinue their participation if the rewards are perceived to be of lesser value than the cost of participating. From the activity perspective, both frequency and type of participation are pivotal indicators concerning well-being and quality of life [32]. For instance, Silverstein and Parker’s [5] study revealed that leisure participation is positively related to reported leisure and life satisfaction. In general, researchers agree that regular participation in leisure activities is good for individual health and well-being. Conversely, withdrawing from routine leisure activity is likely to negatively impact individuals and such negative impacts are particularly significant for elderly adults [35]. In the longitudinal study, Agahi et al. [36] pointed out that elderly adults may start new leisure activities and cease previous involvements due to a decrease in their physical abilities and functional status. In this study, leisure participation refers to the behavior of TCC exercise. Since the respondents of the study were elderly adults, participation in TCC exercise has become one of major leisure activities in their current lives. Most importantly, elderly adults are recommended to participate in activities with moderate strength [35]. The TCC is one of the moderate-strength leisure activities particularly suitable for elderly adults.

## 3. Hypothesis Development

In developing hypotheses for testing, this section addresses whether psychological well-being is related to leisure participation behavior, leisure participation behavior is associated with antecedent variables (i.e., attitude, subjective norm, perceived behavioral control) and whether these variables are related to designed belief constructs and applied TAM variables (i.e., perceived ease of use, perceived usefulness). Previous studies linked to the proposed variables are also discussed.

### 3.1. Leisure Participation Behavior and Psychological Well-Being

The psychological benefit of leisure participation is a popular topic in the field of leisure study [5]. This fact is due to the belief that the psychological benefit is considered a useful evaluation criterion related to leisure activity participation [37] and is correlated with perceived quality of life [38]. Through leisure participation behavior, individuals are able to build social networks, obtain new knowledge/skills, perform positive feelings, and, therefore, improve individual psychological well-being. Conceptually, leisure participation is a behavior that can increase one’s health and psychological well-being. That is, leisure participation may be an antecedent in helping individuals experience psychological well-being [39]. In the literature, regular exercise behavior has been characterized as a positive leisure participation that may yield psychological benefits [40]. It is believed that TCC practices are beneficial for elderly adults because TCC offers various advantages (e.g., relax). Research has also shown that a positive relationship between leisure activity participation and psychological well-being exists [8,9,39]. 

By evaluating and referencing the results of prior studies, the research team for this study believe that participation in TCC practices is beneficial for elderly adults. This is because TCC offers exercise for the mind as well as for the body [41]. Through such leisure activity, elderly adults may view this exercise as a means for maintaining an active mind as well as pursuing physical fitness. In summary, both mind and body can be exercised or activated through the participation in TCC practices. As previously stated, research has shown that the relationship between leisure participation behavior and psychological well-being is positive and, as such, has led to the following hypothesis:

**Hypothesis 1** **(H1).** *Elderly adults’ TCC participation behavior positively influence their psychological well-being.*

### 3.2. Attitude and Leisure Participation Behavior

The TPB model proposes that attitudes toward a particular behavior, subjective norm, and perceived behavioral control are independent components concerning behavioral intention. As one of the volitional factors, attitude is defined as “the degree to which a person has a favorable or unfavorable evaluation or appraisal of the behavior in question” [21]. Attitude is a function of an individual’s behavioral beliefs (BB). More specifically, BB is one’s perceived subjective significance that exercising can lead to certain results [20]. In this case, elderly adults may perceive that TCC can be helpful for their health so that they demonstrate positive attitudes toward this particular behavior. If a person decides to perform a particular behavior, he/she tends to assess whether the benefit resulting from such behavior can outweigh the cost [42]. In general, an individual is likely to have a positive attitude if the outcome evaluations and results are known and positive. As a result, this individual is likely to perform, and repeat, that particular behavior [21,42], which leads to the following hypothesis:

**Hypothesis 2** **(H2).** *Attitude positively influences elderly adults’ TCC participation behaviors.*

### 3.3. Subjective Norm and Leisure Participation Behavior

As another non-volitional factor in TPB, subjective norm is defined as “the perceived social pressure to perform or not to perform the behavior” [21]. More specifically, subjective norms refer to the perceived beliefs or impressions of significant others who are closely associated with that specific individual (e.g., spouse, mentor, family members, friends, colleagues) and who impact his/her decision making process about whether he/she should engage in a particular behavior [24,43]. Conceptually, subjective norm is a function of an individual’s normative beliefs about what significant others’ thoughts about acceptable and unacceptable behaviors. In the TPB model, subjective norms determine behavioral intention, which is well-documented in the leisure literature [44,45,46]. Thus, in this case, if significant others believe that TCC is an appropriate behavior and it is good for psychological well-being, an individual’s perceived social pressure to perform TCC may increase their motivation to conform to the preferences or opinions of their social networks, which leads to the following hypothesis:

**Hypothesis 3** **(H3).** *Subjective norms positively influence elderly adults’ TCC participation behaviors.*

### 3.4. Perceived Behavioral Control and Leisure Participation Behavior

Perceived behavioral control is the third determinant related to behavioral intention and an added factor to address a non-volitional situation. Ajzen [21] defines perceived behavioral control as “the perceived ease or difficulty of performing the behavior.” Han et al. [22] noted that perceived behavioral control assesses individual perception concerning one’s control over situational factors that may either facilitate or restrain that behavior. Perceived behavior control is described as a function of control beliefs that refers to one’s assessment in resource/opportunity availability. If resources and/or opportunities are present, one would be likely to engage in that particular behavior, and vice versa. In this study’s context, if a field for TCC exercise is easy to access and the cost of joining a TCC practice is reasonable, the probability of elderly adults who are willing to perform TCC would increase. Conversely, if a field for TCC inconveniently located and joining group practices is expensive, only a few elderly adults may choose to participate in this leisure activity. The following hypothesis is developed:

**Hypothesis 4** **(H4).** *Perceived behavioral control has a positive influence on elderly adults’ TCC participation behavior*.

### 3.5. Perceived Ease of Use, Perceived Usefulness, and Attitude

Davis [16] posits that the constructs of perceived ease of use and perceived usefulness are the cornerstones of users’ attitudes toward using a new system. Perceived ease of use is defined as “the degree to which a person believes that using a particular system would be free of effort,” while perceived usefulness is defined as “the degree to which a person believes that using a particular system would enhance his or her job performance” [16]. The two constructs are built from the user’s perspective. More specifically, if using a specific product or system requires little effort and it is considered to be helpful to a user’s performance in a given task, he/she is likely to use it and vice versa. In this study, perceived ease of use refers to the degree to which an elderly adult believes that TCC will require them to expand little effort. Perceived usefulness refers to the degree to which an elderly adult believes that TCC is a helpful leisure activity that aids health and relaxation.

Past research on perceived ease of use and individual attitude indicates a positive relationship between the two constructs [16,23]. Studies on perceived usefulness and individual attitude also reveal a positive relationship [16,23]. Similar results were also found in the fields of sports, leisure, and recreation [47,48]. In this study’s context, if elderly adults perceive that TCC is not difficult to learn and perform, they are likely to form a positive attitude toward such behavior. If elderly adults perceive that TCC is useful for keeping their mind sharp or maintaining their physical fitness, they are likely to have a positive attitude toward this particular behavior. Therefore, the following hypotheses were developed:

**Hypothesis 5** **(H5).** *Perceived ease of use is positively associated with elderly adults’ attitudes toward TCC participation behavior.*

**Hypothesis 6** **(H6).** *Perceived usefulness is positively associated with elderly adults’ attitudes toward TCC participation behavior.*

### 3.6. Normative Belief and Subjective Norm

Subjective norms are regarded as a function of salient normative belief [49]. In TPB, normative norm refers to an individual perception in terms of social pressures or the beliefs of other people that a person should or should not perform a particular behavior [50]. Motivation to comply is defined as a person’s choice of whether such person follows instructions and meets outcomes preferred by important referents [50]. Basically, normative belief is a concept of social influence. Such social influence refers to accepted standards or unwritten rules of behavior, which take place in a particular group, community, or culture. It can be said that important referents in combination with the individual’s motivation to comply is comprised of the prevailing subjective norms. Each individual has salient groups or referents around him or her. Salient groups or referents surround individuals and these groups may contain family members, friends, mentors, or coworkers. Individuals are more likely to perform certain behaviors when they are aware of referents’ preferences in terms of a particular behavior [49]. In our context, if salient referents are able to encourage and offer suggestions to elderly adults by pointing out that practicing TCC is good for them, they are likely to follow referents’ suggestions to participate in TCC activities. In this study, the important referents included family members and friends. Walen and Lachman [51] and Galleant et al. [52] indicated that family members and friends are generally the most impactful individuals in shaping a particular person’s point of views. Therefore, the following hypotheses were developed:

**Hypothesis 7** **(H7).** *Family members’ influence is positively associated with subjective norms as perceived by elderly adults’ decision in participating in TCC practice.*

**Hypothesis 8** **(H8).** *Friends’ influence is positively associated with subjective norms as perceived by elderly adults’ decision in participating in TCC practice.*

### 3.7. Control Belief and Perceived Behavior Control

In TPB, perceived behavior control is considered a function of control belief. Control belief refers to the presence of factors that may either promote or obstruct the performance of a specific behavior [53]. That is, if the participation of an activity is convenient (e.g., easy access, low cost) for a person, this particular person is more likely to participate in that activity, and vice versa. Enabling or hindering conditions may play a pivotal role in whether a person is willing to participate in a leisure activity or not. Ajzen [21] addresses that perceived behavioral control can be considered the concept of self-efficacy. Self-efficacy refers to the belief in an individual’s capabilities of “how well one can execute courses of action required to deal with prospective situations” [54]. Also, self-confidence in a person’s ability is another key to an individual performing a particular behavior [55]. Self-efficacy influences the individual selection of activities, and it can also affect determination and the amount of effort expanded during the execution process [21]. In summary, control belief is comprised of the internal force of self-efficacy and the external factor of facilitated conditions. In this context, if elderly adults perceive that they are confident in TCC and they are able to perform well, they are likely to engage in an increased effort and dedicate time for this activity. If doing TCC practice can be cost efficient, based on a person’s time availability, as well as conveniently located, elderly adults are likely to become involved in the leisure activity. Researchers [56,57] have indicated that both self-efficacy and facilitation conditions are positively associated with perceived behavioral control. Therefore, the following hypotheses were developed:

**Hypothesis 9** **(H9).** *Self-efficacy is positively associated with perceived behavioral control as perceived by elderly adults.*

**Hypothesis 10** **(H10).** *Facilitation conditions are positively associated with perceived behavioral control as perceived by elderly adults.*

**Figure 3:**
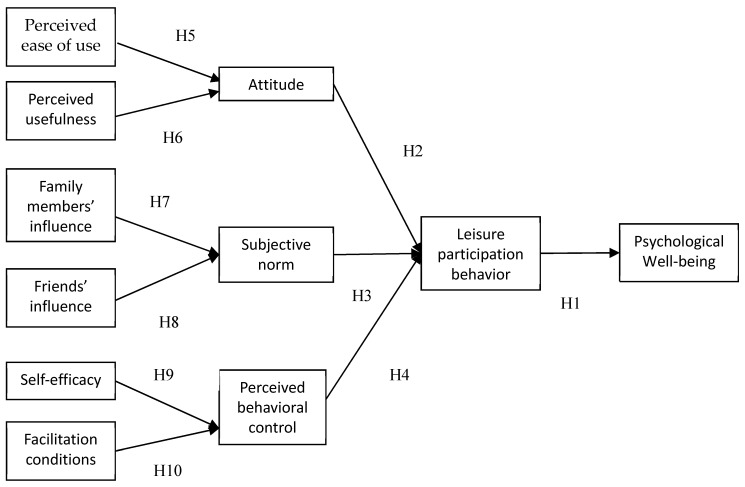
Concept of research model.

Summarized and integrated the hypotheses development above, Figure 3 illustrates the model for the present study. 

## 4. Materials and Methods

### 4.1. Measurement

To effectively assess respondents’ cognitive performance, the development of the measurement was based on theories, an extensive review of the literature related to psychological well-being and leisure participation behavior, as well as structured interviews with five individuals who conduct TCC practices routinely. The construct of perceived ease of use was measured by four statements adopted from Davis [16] and Cardinal [58]. The construct of perceived usefulness was measured using six statements adopted from Davis [16], Cardinal [58], and Venkatesh and Davis [59]. Normative belief was grouped into two dimensions including family members’ influence and friends’ influence. Both dimensions were measured using four statements adopted from Taylor and Todd [55], Downs and Hausenblas [60], and Curtis et al. [61]. The construct of control belief includes self-efficacy and facilitation conditions. Self-efficacy was measured by four statements adopted from Bandura [62]. Facilitation conditions were measured using three statements. The development of these was based on Ajzen [21] and Taylor and Todd [55]. Drawing on previous studies [50,63], attitude was measured by five statements. Based on Taylor and Todd [55], Downs and Hausenblas [60], and Curtis et al. [61], three statements were developed to measure the construct of subjective norm. Perceived behavior control was measured by three statements adopted from Ajzen [21] and Taylor and Todd [55]. Four statements were used to measure the construct of leisure participation behavior. The development of these statements was centered on the works of Fishbein and Ajzen [50]. A total of eight statements were used to measure the construct of psychological well-being. The development of these statements were adopted from Ajzen [21], Pouwer et al. [64], Hills and Argyle [65], and Chang et al. [3]. Totally, 48 items used for measuring eleven constructs are presented in Table 1. Concerning the response categories of this study, a 7-point Liker-type scale (ranging from 1 = strongly disagree to 7 = strongly agree) was employed to measure all variables of this study.

A pilot test with 98 individuals who participated in TCC practice was conducted in order to verify the wording, ease in responding, and applicability of statements [66]. This study made some modifications to the wording of statements as a result of the pre-test. The survey included items worded with proper negation and a randomization to reduce the monotony of items measuring the same construct. The items were developed bilingually, in Chinese and English, considering the linguistic difficulties of interpreting problems and to prevent misleading the participants.

### 4.2. Data Collection and Sample Profile

Purposeful sampling was employed in this study because a complete population list of elderly adults who performed TCC was unavailable or impossible to obtain. Participants were elderly adults who performed TCC practice aged 60 years or above. Face-to-face administration was employed in this study. The data collection method was approved by the research review committee of investigators’ affiliated institution. Respondents were informed that their responses to the questionnaire were absolutely voluntary. The period of data collection lasted near two months from 5 July 2018, to 28 August 2018. As a result, a total of 769 usable responses were collected and used in the data analysis. 

Of the 769 respondents, 58.3% were male (*n* = 448). Female respondents accounted for 41.7% (*n* = 321). Respondents’ ages ranged from 60 to 86 years. The average age was 69.7 years. Respondents who had graduated from middle school (33.4%, *n* = 257), high school or vocational school (29.5%, *n* = 227), and college (25.1%, *n* = 193) were the major categories of education level. The majority of the respondents indicated that they currently lived with their family members (74.9%, *n* = 576). Approximately 67.4% of the respondents (*n* = 518) revealed that they practiced TCC for at least a year. Among them, about 47.3% of respondents (*n* = 364) reported at least five years of experience in TCC.

## 5. Results of Analysis

### 5.1. Descriptive Statistics

The descriptive statistics were analyzed by SPSS 22 software, the results of which are listed in Table 1. On average, the elderly adults’ participation of TCC was responded positively (the mean of every construct is greater than 4.99 out of 7).

### 5.2. Construct Validity and Reliability

Researchers developed the structural equation model (SEM), a powerful two-step multivariate technique for analyzing the causal models, to evaluate the degree that proposed conceptual model containing observed multiple indicators and hypothetical constructs explained or fit the collected data. The SEM statistic is a multivariate technique that combines factor analysis and multiple regressions in addition to enabling the researchers to assess a series of independent/dependent relationships simultaneously [67]. Such analytical techniques have been widely applied in recent years. This study utilized the SEM to empirically test the relationships between constructs using the AMOS 22 software program. According to Anderson and Gerbing [68], to test and estimate the hypothesized model, this work employed a two-step approach with an initial measurement model and a subsequent structural model.

The measurement model uses confirmatory factor analysis (CFA) to determine whether the constructs are sufficiently valid and reliable. Thus, before testing the proposed model, the data were examined for the purpose of screening any violation of assumptions concerning the linear model. CFA was performed to assess the underlying structure of the proposed model. Table 1, the results of CFA, presents an overview concerning the means, standard deviations, and correlation among the constructs. The standardized loadings of all statements ranged from 0.55 to 0.92 on their proposed constructs which exceeds the minimum hurdle level of 0.50 recommended by Hair et al. [67]. These results indicated that the statements in the questionnaire were significantly associated with their specified constructs and each scale’s unidimensionality was satisfactory. Composite reliability of the underlying constructs ranged from 0.78 to 0.95 and the values exceeded the suggested value of 0.70, which was recommended by Bagozzi and Yi [69]. Average variance extracted (AVE) was also employed for the purpose of examining the convergent validity of the measures. The AVE values ranged from 0.545 to 0.831 and exceeded the recommended value of 0.50 suggested by Fornell and Larcker [70]. In addition, the square root values of AVE for the measured construct were larger than the correlation between each construct. These values indicated that discriminant validity was satisfactory. Table 2 presents an overview concerning the correlations, reliability coefficients, and AVEs. The foregoing analysis shows that the measurement model tests, including convergent and discriminant validity and reliability measures, are satisfactory.

### 5.3. Tests of Structural Model

In this study, the SEM technique is an appropriate tool for measuring parametric values (i.e., path coefficients) for each of the research hypotheses implemented on the basis of the TPB and the TAM to determine their respective significance. After assessing the measurement model, we evaluated an initial theoretical model having seven constructs with six gamma paths and four beta paths. As the first step in assessing the hypothesized relationships, the structural equation model was evaluated by examining the χ^2^ and fit indices. The performed fit indices included the goodness of fit index (GFI), Adjusted goodness of fit index (AGFI), root mean square error of approximation (RMSEA), comparative fit index (CFI), and normed-fit index (NFI). The results indicated that χ^2^/df = 2.328, which is less than the standard score of 3 suggested by Bentler and Bonett [71]. The GFI had a score of 0.811 exceeding the score of 0.8 recommended by Hair et al. [67]. The AGFI has a score of 0.805 exceeding the score of 0.8 recommended by Scott [72]. The RMSEA had a score of 0.069, and met the requirement (less than 0.8) suggested by Hair et al. [67]. The scores of the CFI (0.912) exceeded 0.9, while NFI score (0.845) was a little lower than 0.9 recommended by Brown and Cudeck [73]. Thus, these indicators consistently show an acceptable fit between the hypothesized model and the data.

**Figure 4:**
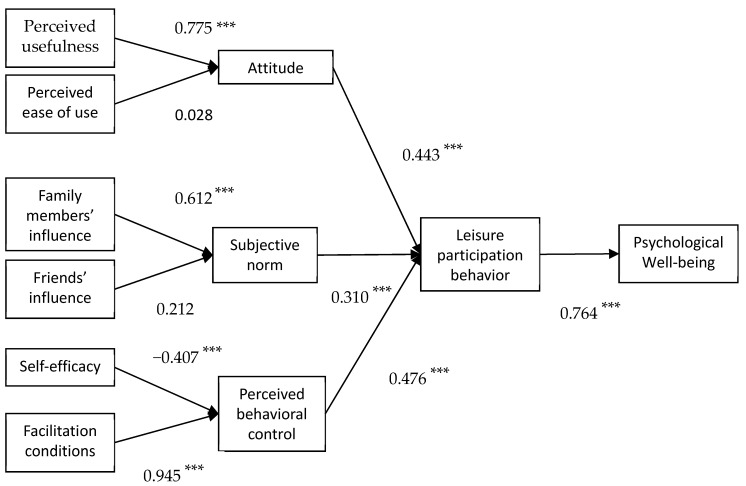
Results of testing hypothetical model (N = 769). *** *p* < 0.001.

The results of hypothesis testing are presented in Figure 2. The estimates of the standardized coefficients indicated that the linkages between leisure participation behavior and psychological well-being (β = 0.764, *p* < 0.001), between attitude and leisure participation behavior (β = 0.443, *p* < 0.001), between subjective norm and leisure participation behavior (β = 0.310, *p* < 0.001), between perceived behavioral control and leisure participation behavior (β = 0.476, *p* < 0.001) were all positive and significant. Hypotheses 1, 2, 3, and 4 were, therefore, supported. The linkage between perceived usefulness and attitude (β = 0.775, *p* < 0.001) was positive and significant. However, the linkage between perceived ease of use and attitude was not significant. Thus, Hypothesis 5 was supported. Hypothesis 6 was not supported. The linkage between family members’ influence and subjective norms was significant (β = 0.612, *p* < 0.001), but the linkage between friends’ influence and subjective norms was not significant. Hypothesis 7 was supported. Hypothesis 8 was not supported. Finally, the linkage between facilitation conditions and perceived behavioral control (β = 0.945, *p* < 0.001) was significant. Although the linkage between self-efficacy and perceived behavioral control (β = −0.407, *p* < 0.001) was significant, the direction of the relation was not predicted. Therefore, hypothesis 9 was not supported, whereas Hypothesis 10 was supported. The findings for these hypotheses showed that elderly adults’ psychological well-being is positively correlated with their participation in TCC practice behavior, and their attitude toward TCC, subjective norms, and perceived behavioral control all seemed to impact their participation behavior. In addition, as shown in Figure 4, the estimates of the standardized coefficients indicated that the effect of perceived behavioral control on leisure participation behavior was greater than attitude and subjective norm. The effect of perceived usefulness on attitude was greater than perceived ease of use. The effect of family members’ influence on subjective norm was greater than friends’ influence. Finally, the effect of facilitation conditions on perceived behavioral control was greater than self-efficacy.

## 6. Discussion and Implications

### 6.1. Discussion

In regard to measurement instrument, the empirical results of confirmatory factor analyses coincide with the research findings of previous studies including Davis [16], Cardinal [58], Venkatesh and Davis [59], Taylor and Todd [55], Downs and Hausenblas [60], Curtis et al. [61], Bandura [62], Ajzen [21], Pouwer et al. [65], Hills and Argyle [64], and Chang et al. [3]. This finding enhances the applicability of the scale in the leisure participation behavior and psychological well-being setting. However, even the reliability of subjective norm (0.89) exceeded the minimum hurdle level of 0.70 and the variance extraction measure was 0.615, which also exceeded the minimum hurdle level of 0.50 recommended by Hair et al. [67]. Although the squared correlation with leisure participation behavior was 0.8, which is higher than the square root of AVE (0.708), this statistic did not meet the requirement of a conservative test of discriminant validity. This result suggested that the measure of subjective norms and leisure participation behavior do not actually capture a distinct or isolated trait. Thus, the result suggested that these two constructs are suitable for further investigation.

On the other hand for the hypothesized model, by combining TAM as a part of the TPB model, this study endeavored to test the appropriateness of TPB in explaining the elderly adults’ decision making process concerning leisure participation behavior and the effect of engaging in the behavior on individual psychological well-being. Overall, the results of the study verified that the proposed constructs can be the main reasons for elderly adults’ participation in TCC practice behavior as well as the possibility of this behavior contributing to their psychological well-being. The results are consistent with the findings, attitudes toward a particular behavior, subjective norms, and perceived behavioral control are independent components concerning leisure participation behavior, studied by Han [13], Lee [14] and Weimann et al. [15], and also consistent with the findings that leisure participation and perceived well-being are all positively related as shown by Silverstein et al. [5], Netz et al. [6], Ku et al. [9], Vozikaki et al. [8] and Jopp et al. [39]. According to the results for TCC, elderly adults may become happy, confident, and satisfied with their current quality of life. Furthermore, the findings of the study also verified the roles of antecedent variables, which consisted of belief constructs (i.e., family members’ influence, facilitation conditions) and TAM applied constructs (i.e., perceived usefulness), to specifically explain the path of the proposed model. The results showed one weak relation, one non relation, and one relation that was the opposite of the predicted relation, which did not support the hypotheses. 

The path between perceived ease of use and attitude (H6) is weak and insignificant. Therefore, perceived ease of use providing utilitarian value does not necessarily lead to a different attraction and facilitation in TCC participation. For TCC participation and practice, an elderly adult needs to understand the practice rules and skills that must be learned from a mentor or read from a guidebook. Elderly adults may assess the TCC guidebook for practicing and/or mentors’ teaching styles, which may not be easy to understand. Therefore, improvements are necessary. The relationship between friends’ influence and subjective norms (H8) is also weak and not significant. Since salient groups or referents include family members, friends, mentors, or coworkers, most elderly adults maybe feel that they encourage being accompanied and offer suggestions from family members is enough. Therefore, friends’ influence is neglected and insignificant. Finally, the relationship between self-efficacy and perceived behavioral control (H9), was the opposite of the predicted hypothesis. This observation suggests that elderly adults perceive that they are confident in executing TCC, but they are unable to perform it well, possibly because elderly adults think they are old, their physical strength and cognitive abilities have declined, and TCC is a new leisure activity for them, the guidebook for practicing is not easy to understand, and/or mentors’ teaching style is not clear. Therefore, individuals cannot practice TCC well through individual practices. Furthermore, she/he will feel embarrassed if she/he unable to perform TCC well and handle any miscues related to TCC. Moreover, in Chinese culture, elderly adults are ashamed to ask someone for help. 

### 6.2. Implication

This study offers theoretical implications for a better understanding of the determinants of decision making processes concerning elderly adults’ leisure participation behavior (i.e., TCC practice behavior). Although some limitations existing in the hypothesized model analyses, the major findings of this study have significant managerial implications for health policy makers and/or clinical interventions to use these participations’ behavior relationships. First, the findings indicated that elderly adults’ TCC practice behavior significantly affects their psychological well-being. More so, this finding also implies that through participation in leisure activities in general, individuals may improve psychological well-being. Additionally, elderly adults may become happy, confident, and satisfied with their current quality of life. Thus, health policy makers in government and/or clinical interventionists can use TCC participations as an effective way to promote well-being in elderly adults since the use of TCC to enhance and maintain psychological well-being for elderly individuals has two potential advantages [74]. First, TCC is a low-cost, safe and easily implemented leisure activity that is performed at a low to moderate intensity. Second, TCC requires minimal staff and equipment and it can be adopted effectively as a simple community-based intervention. Thus, TCC is a simple slow-movement leisure activity, suitable for elderly individuals with diminished physical functioning [75].

Second, one can emphasize the usefulness of TCC practice to attract and facilitate elderly adults’ participation. When engaging in a particular leisure activity (i.e., TCC), elderly individuals should assess the usefulness, as well as the ease of both starting and comprehension of an activity. In this case, the finding revealed that perceived usefulness had a greater level of impact on attitude. Such results imply that elderly individuals need to realize the usefulness of TCC practice as mildly demanding, both physically and mentally, tasks that are perceived positively, which increase the likelihood that individuals will start and continue the exercise.

Third, the TCC program should set in convenient locations. The findings of this study indicated that non-volitional variables play a pivotal role concerning elderly adults’ decision making process in TCC. Perceived behavioral control (β = 0.476, *p* < 0.001) had the strongest influence on leisure participation behavior (i.e., TCC exercise behavior) than attitude (β = 0.310, *p* < 0.001) and subjective norm (β = 0.443, *p* < 0.001). These results imply that elderly adults are more likely to engage in TCC if they have spare time, feel economically sufficient, and consider convenient locations for practicing this leisure activity. Surprisingly, the findings of this study revealed that elderly adults did not have confidence in performing TCC well, but they would put forth time and great effort for this activity. 

Fourth, elderly adults’ participation rates can be enhanced via social promotion. The findings of this study also revealed that family members’ influence is the primary factor to impact individual decision making for engaging in TCC. A possible explanation is that a majority of respondents’ family members may consider TCC as a good leisure activity. In addition, the encouragement to participate in TCC practice can be good for respondents personally as well as relationally since TCC can be an exercise performed by a group of people and, therefore, respondents have opportunities for interactions with others individuals as an integral part of the leisure activity. Prior research also show that better self-reported individual health is positively correlated with the number of leisure activity events [3,37,76]. 

However, several limitations do exist in this study. First, purposeful sampling was used in this study because investigators were unable to obtain a complete population list. Thus, the results of the study cannot be generalized to all individuals who practice TCC. In addition, the results of the study cannot be generalized to Western countries, as TCC is deeply rooted in Asian perspectives and values. Second, the respondents of this study were exclusively elderly adults who practiced TCC. The results of the study are not applicable for leisure activities other than TCC. Finally, the levels of TCC activity can be varied based on skill sets and years of experiences in practice. Therefore, the perception of performing TCC as easy or difficult can be different. A TCC practitioner with several years of experience, using perceived ease of use as an example, may regard this leisure activity as an easy task, however, it may not be an easy task for a novice. This topic has not been fully addressed but it does provide researchers with a direction for future investigations into this topic area. Moreover, this study did not consider the relationship between the factors, such as TCC styles, frequency of practice, and duration of each session, with the health effects. Hence, another area for exploration is a scoring framework to evaluate whether different TCC styles, frequency of practice (per week), duration of each session (minutes), interfere with the health effects and quality of life. 

## 7. Conclusions and Suggestions for Future Research

This paper takes an effort to develop and examine a theoretical explanation regarding the formation of elderly adults’ psychological well-being and participation in TCC exercise. The results indicated that perceived behavioral control, attitude, and subjective norm are positively and significantly related to TCC participation behavior. TCC participation behavior has positive and significant effects on elderly adults’ perceived psychological well-being. Perceived usefulness has positive and significant effects on respondent attitude. The results also showed that family members’ influences is positively and significantly related to participants’ subjective norms, resource-facilitating conditions are positively and significantly related to perceived behavioral control. 

This study explored external variables that influence attitude, and subjective norm and perceived behavioral control in participating TCC, and ultimately bring elderly adults’ perceived psychological well-being in China with regard to TPB constructs. Thus, from an academic viewpoint, the results of the study are consistent with evidence in the general TPB and contribute to the leisure and aging literature, and develop a theoretical reference model for a better understanding the leisure participation perceptual reasoning processes of elderly adults. We aim to initiate a new theoretical perspective for research in the field of leisure participation behavior and well-being of elderly adults, from practitioners’ viewpoints, the proposed model can provide health makers and/or clinical interventionists with a theoretical model, and professionals with information for building well-constructed leisure program consumers’ perspective. In other words, the study establishes the representative dimensions of desirable leisure programs as well as the relevant indicators that measure each dimension. The indicators constitute a valid and reliable measurement instrument. Thus, such a scale can serve as a managerial tool to the extent that program providers can further evaluate program performance and initiate proper practices, which are aimed at improving the services provided and inspiring participation and, hence, improving the psychological well-being of elderly adults.

## Figures and Tables

**Table 1:** Descriptive statistics (mean and standard deviation (SD), *N* = 769).

Variable	Mean	SD	Loading	CR
Perceived ease of use				
I feel Tai Chi Chuan (TCC) is an activity which is easy to learn.	5.98	1.36	0.90	
I feel the guidebook for TCC practice is easy to understand.	5.66	1.30	0.92	0.95
I feel the rules of TCC practice is easy to understand.	5.92	1.25	0.92	
I feel the skills required for TCC practice is easy.	5.97	1.15	0.91	
Perceived usefulness				
I feel practicing TCC helps improve my physical health.	6.09	1.12	0.76	
I feel practicing TCC helps me stay away from loneliness.	5.95	1.27	0.75	
I feel practicing TCC provides opportunities to interact with others.	5.02	1.61	0.82	0.91
I feel practicing TCC helps increase the enjoyment of my life.	5.07	1.53	0.82	
I feel practicing TCC helps improve my psychological health.	5.07	1.56	0.80	
I feel practicing TCC helps improve my ability to think.	5.14	1.57	0.77	
Family members’ influence				
My family members encourage me to participate in TCC practices.	5.55	1.29	0.86	
My family members would accompany me to participate in TCC practices.	5.18	1.55	0.67	0.88
My family members recommend that I engage in TCC practices when possible.	5.50	1.36	0.89	
My family members think that TCC is a good leisure activity for me.	5.72	1.26	0.81	
Friends’ influence				
My friends encourage me to participate in TCC practices.	5.57	1.32	0.87	
My friends would accompany me to participate in TCC practices.	5.32	1.50	0.83	
My friends recommend that I engage in TCC practices when possible.	5.56	1.29	0.91	0.91
My friends think that TCC is a good leisure activity for me.	5.74	1.24	0.76	
Self-efficacy				
I am confident that I am able to practice TCC well if a practice guide is available.	5.16	1.55	0.87	
I am confident that I am able to practice TCC well if someone else can provide instructions.	5.30	1.38	0.81	0.92
I am confident that I am able to handle any miscues related to TCC.	5.12	1.50	0.90	
I am confident that I am able to practice TCC well through individual practices.	5.20	1.49	0.85	
Facilitation condition				
I have a plenty of spare time to practice TCC.	5.30	1.42	0.74	
I have sufficient economic resources to practice TCC.	4.99	1.67	0.73	0.78
A field for TCC practices is conveniently located near my place.	5.39	1.37	0.75	
Attitude				
Practicing TCC is beneficial to me.	6.03	1.18	0.87	
Practicing TCC is a pleasant experience to me.	5.97	1.17	0.88	
Practicing TCC is good for me.	6.06	1.16	0.92	0.94
The experience of practicing TCC is meaningful to me.	6.01	1.16	0.85	
I am interested in practicing TCC.	6.11	1.13	0.84	
Subjective norm				
Many of my friends engage in TCC practices.	5.43	1.46	0.84	
My family members are supportive to my engagement in TCC practices.	5.57	1.28	0.82	0.89
I care about suggestions concerning participation in TCC practices.	5.67	1.29	0.90	
Perceived behavior control				
I have sufficient knowledge/ability in TCC.	5.36	1.47	0.85	
I am capable of becoming a good TCC player.	5.46	1.32	0.86	0.90
I think I am in control of participating in TCC practices.	5.51	1.39	0.87	
Leisure participation behavior				
I practice TCC routinely.	5.40	1.46	0.82	
I have been practice TCC all along.	5.54	1.43	0.86	
I will continue to practice TCC in the future.	5.62	1.35	0.87	0.86
I will participate in TCC competitions.	5.15	1.69	0.55	
Psychological well-being				
Practicing TCC makes me feel a sense of fulfillment.	5.86	1.24	0.84	
Practicing TCC makes me feel satisfied.	5.92	1.20	0.86	
Practicing TCC makes me enjoy the moment of engaging in such activities.	5.94	1.17	0.88	
Practicing TCC increases my feeling of optimism.	6.00	1.15	0.90	0.95
Practicing TCC makes me content with my life.	6.09	1.15	0.88	
Practicing TCC enriches my individual feeling.	6.90	1.23	0.77	
Practicing TCC makes me happy.	5.92	1.18	0.80	
Practicing TCC increases my confidence.	5.99	1.19	0.80	

CR = Composite Reliability.

**Table 2:** Measure of correlations, reliability coefficients, and average variance extracted (AVE).

	PEOU	PU	FMI	FI	SE	FC	ATT	SN	PBC	LPB	PWB	AVE
PEOU	0.91											0.831
PU	0.73	0.79										0.619
FMI	0.75	0.45	0.81									0.657
FI	0.77	0.50	0.73	0.84								0.709
SE	0.71	0.78	0.38	0.42	0.86							0.733
FC	0.61	0.43	0.61	0.70	0.24	0.74						0.545
ATT	0.83	0.57	0.70	0.70	0.51	0.54	0.87					0.761
SN	0.63	0.40	0.78	0.81	0.34	0.56	0.58	0.85				0.729
PBC	0.49	0.36	0.49	0.55	0.22	0.68	0.43	0.44	0.86			0.737
LPB	0.77	0.52	0.77	0.70	0.43	0.69	0.71	0.80	0.59	0.78		0.615
PWB	0.59	0.39	0.59	0.61	0.33	0. 50	0.62	0.61	0.45	0.76	0.84	0.708
CR	0.952	0.907	0.883	0.907	0.916	0.782	0.941	0.890	0.894	0.961	0.951	

Note. Diagonals represent the square root of AVEs. PEOU = perceived ease of use; PU = perceived usefulness; FMI = family members’ influence; FI = friends’ influence; SE = self-efficacy; FC = facilitation conditions; ATT = attitude; SN = subjective norm; PBC = perceived behavioral control; LPB = leisure participation behavior; PWB = psychological well-being; CR = composite reliability.
